# Balance and muscle power of children with Charcot-Marie-Tooth

**DOI:** 10.1590/bjpt-rbf.2014.0055

**Published:** 2014

**Authors:** Tais R. Silva, Amanda Testa, Cyntia R. J. A. Baptista, Wilson Marques, Ana C. Mattiello-Sverzut

**Affiliations:** 1 Curso de Fisioterapia, Faculdade de Medicina de Ribeirão Preto (FMRP), Universidade de São Paulo (USP), Ribeirão Preto, SP, Brasil; 2 Departamento de Biomecânica, Medicina e Reabilitação do Aparelho Locomotor, FMRP, USP, Ribeirão Preto, SP, Brasil; 3 Departamento de Neurociências e Ciências do Comportamento, FMRP, USP, Ribeirão Preto, SP, Brasil

**Keywords:** Charcot-Marie-Tooth disease, strength, balance, range of movement, assessment, physical therapy

## Abstract

**BACKGROUND:**

In certain diseases, functional constraints establish a greater relationship with muscle power than muscle strength. However, in hereditary peripheral polyneuropathies, no such relationship was found in the literature.

**OBJECTIVE:**

In children with Charcot-Marie-Tooth (CMT), to identify the impact of muscle strength and range of movement on the static/dynamic balance and standing long jump based on quantitative and functional variables.

**METHOD:**

The study analyzed 19 participants aged between 6 and 16 years, of both genders and with clinical diagnoses of CMT of different subtypes. Anthropometric data, muscle strength of the lower limbs (hand-held dynamometer), ankle and knee range of movement, balance (Pediatric Balance Scale) and standing long jump distance were obtained by standardized procedures. For the statistical analysis, Pearson and Spearman correlation coefficients were used.

**RESULTS:**

There was a strong positive correlation between balance and the muscle strength of the right plantar flexors (r=0.61) and dorsiflexors (r=0.59) and a moderate correlation between balance and the muscle strength of inversion (r=0.41) and eversion of the right foot (r=0.44). For the long jump and range of movement, there was a weak positive correlation with right and left plantar flexion (r=0.20 and r=0.12, respectively) and left popliteal angle (r=0.25), and a poor negative correlation with left dorsiflexion (r=-0.15).

**CONCLUSIONS:**

The data on the patients analyzed suggests that the maintenance of distal muscle strength favors performance during balance tasks, while limitations in the range of movement of the legs seem not to be enough to influence the performance of the horizontal long jump.

## Introduction

Charcot-Marie-Tooth disease (CMT) is a hereditary polyneuropathy with various subtypes. The common clinical phenotype is the impairment of motor and sensory peripheral nerves due to a demyelinating and axonal degenerative process¹. The predominant distal muscle weakness may cause significant motor dysfunction in ambulation, participation in daily life, socio-cultural activities in both children and adults. It is important to note that weakness of the ankle dorsiflexors occurs in association with shortening of the plantar flexor muscles and the development of foot deformities^2^.

The main clinical hypothesis for the development of foot deformities focuses on the intimate relationship between the imbalance in the strength of the invertor and evertor muscles of the feet and overload of the plantar flexor muscles, in contrast to the weakness of the dorsiflexor group^3^. The latter is considered the main manifestation of the disease and contributes to foot deformity (e.g. pes cavus), ankle contracture, poor motor function and difficulty in walking in affected children and adults^2^.

It is believed that losses in the range of motion (ROM) of distal muscles in patients with CMT compromise muscle power as they impair the stretching-shortening cycle. In the case of the horizontal jump (i.e.standing long jump), 50% of the muscle performance is attributed to the ankle^4^. Thus, the ROM in the lower limbs can be correlated with the performance in a standing long jump test used to infer muscle strength.

Muscle strength, ROM, and different neuromuscular demands in the lower extremity are factors that modify the limits of postural stability and may influence the performance of a specific functional task^5^. Therefore, the selection of physical therapy procedures in CMT disease can be targeted and assertive if based on the understanding of the actual contributions of the variables involved in static and dynamic balance.

It is important to study CMT hereditary polyneuropathy because its incidence is relatively high, affecting 1 in every 2,500 individuals^2^. Although the initial symptoms of the disease usually appear in the first or second decade of life with slow progression over the subsequent decades, adults are the target population in the majority of studies^6^^-^ 8.

Interventional studies involving drugs are still in progress because there is no effective therapy for CMT disease^1^, and the use of orthoses presents controversial results^8^. In addition, studies that focused on clarifying the contribution of the major deficits (i.e. musculoskeletal, neuromuscular, and biomechanical) to balance in children with CMT are limited. Thus, it becomes relevant to investigate the behavior of the biomechanical variables during the initial phase of the disease. This is a preliminary step to the proposal of physical therapy interventions that can potentially help in the rehabilitation of these children and adolescents.

In children and adults, the triad of muscle weakness, joint hyper/hypomobility, and compensatory biomechanical disorders can cause significant motor dysfunctions of distal-proximal predominance with loss of balance, ambulation, and hampered participation in activities of daily living^2^. Similarly, the relationship between passive ROM with horizontal jump, measured using the standing long jump test, and balance, assessed with the Pediatric Balance Scale (PBS), were tested. Briefly, the aim of this study was to evaluate the influence of passive ROM and strength of the major muscle groups of the lower limbs on the static/dynamic balance and horizontal jump capacity of children with CMT disease.

## Method

This study included a total of 19 child and adolescent participants who were admitted to the Neurogenetic Disorders Outpatient Clinic of the *Hospital das Clínicas da Faculdade de Medicina de Ribeirão Preto da Universidade de São Paulo* (HCFMRP/USP), Ribeirão Preto, São Paulo state, Brazil, between 2011 and 2012 with a confirmed CMT diagnoses. The participants were of both genders, aged between 5 and 16 years, were able to walk independently, and had no diseases associated with CMT disease that affect the cardiorespiratory system.

Consent was obtained from the parents or guardians who filled out the informed consent form previously approved by the Research Ethics Committees of the HCFMRP/USP (Number 4334/2011).

In a standardized manner, anthropometric data, goniometry, muscle strength (hand-held dynamometer - Lafayette Instrument Co., Lafayette, UK), lower limb power (long jump test), and static/dynamic balance (Pediatric Balance Scale) were obtained from all participants.

The passive ROM was measured for the knees (popliteal angle) and ankles (plantar flexion and dorsiflexion), according to the method described by Marques^9^. The measurements were performed using a universal goniometer (CARCI - Indústria e Comércio de Aparelhos Cirúrgico e Ortopédicos Ltda.).

The muscle strength (in kilogram-force) of the hip extensors, knee extensors, and dorsiflexors, plantar flexors, supinators, and pronators of the foot were measured three times using a hand-held dynamometer, alternating between the right and left lower limbs to prevent fatigue. The highest value was used for analysis. To ensure that the dynamometer was kept perpendicular to the segment being tested and as distal as possible, an assistant stabilized the participant during the measurements, and the following bodily positions were adopted: supine, lower limbs in the anatomical position and feet out of the stretcher to measure the muscle strengths of the dorsiflexors, plantar flexors, supinators and pronators; the prone position with knee flexed to 90° to measure the muscle strength of the hip extensors; and the sitting position with knee flexed to 90° to measure the muscle strength of the knee extensors. The voice command "force" was used during the tests while the evaluator prevented any range of motion to ensure an isometric contraction for five seconds.

The standing long jump test, also called the horizontal jump or broad jump, is easy to apply and requires only chalk or pencil to mark the ground and a plastic tape measure or self-retracting tape measure to measure the distance jumped. The participants were positioned behind a line marked on the ground with the feet slightly apart and were asked to jump the greatest horizontal distance possible by bending the legs and using the impulse generated by swinging the arms^10^. This strategy allowed balance to be restored or maintained through the transfer of angular momentum from the arms to the rest of the body. Three attempts were made, and the highest value was used for analysis. The result was given in centimeters, measuring the distance between the starting line and the mark achieved by the calcaneus on the ground.

The PBS was used to measure functional balance because it was suitable for school-aged children with mild to moderate motor disability, according to Franjoine et al.^11^. The test lasted approximately 15 minutes and did not require the use of specialized equipment, and it provided clinical data for the measurement of functional balance tasks. The brazilian version of the PBS described by Ries et al.^12^ was used to apply the test. The following materials were used: a chair with back support, adjustable height, and arm rests; markers for the feet; stopwatch; tape measure; and step stool. The participants were instructed, through demonstrations, how they were to perform the tests. A preliminary trial of each proposed task was allowed for each tested item.

The PBS consisted of 14 items that required the child to perform static and dynamic balance tasks. Each item was be scored from 0 to 4, with 4 corresponding to a better ability to perform the required task. The scores on each of the 14 tasks were summed, and the final score was determined from this number, with a maximum value of 56. Higher scores were associated with greater ability to perform the required task and therefore with better balance. In healthy children from the age of seven, the maximum score of 56 should normally be achieved, and there is no mention in the literature regarding the classification of lower scores^11^.

To meet the study objective, which was to correlate the dynamometry data of lower limbs with balance and range of motion data of the lower limbs with the horizontal impulse measured by the standing long jump test, the Pearson correlation coefficient (*r*) and the Spearman correlation coefficient, which quantify the association between two quantitative variables, were used. These coefficients ranged from -1 and 1. A value 0 of (zero) indicated that there was no linear correlation; 1 indicated perfect linear correlation; and -1 also indicated a perfect negative linear correlation (i.e., when one of the variables increased, the other decreased). Values closer to 1 or -1 indicated stronger linear correlation between the two variables. The classification of the Spearman correlation coefficients was performed based on the study by Hulley et al.^13^, and the classification of the Pearson correlation coefficients was performed based on the study by Pagano and Gauvreau^14^. The following correlations were tested: muscle strength × balance and, standing long jump × ROM.

## Results

The anthropometric data and the classification of the participants are shown in Table 1. Among the 19 patients in the study, nine were males and ten were females; the mean age was 10.11 years (standard deviation: 2.64), mean weight was 40.59 kg (standard deviation: 15.37), and mean height was 1.43 m (standard deviation: 0.18). Considering the normative values provided by the World Health Organization (WHO)^15^, nine participants had a body mass index (BMI) appropriate for their age, while four participants were underweight, two were overweight, and four were obese.

**Table 1:** Anthropometric data and classification of participants according to the type of CMT.

Age (years)	Participant	Gender	Weight (Kg)	Height (m)	BMI	Type of CMT
6	**A**	F	36.1	1.3	20***	CMT 1A
6	**B**	F	20.2	1.2	14.5	CMT 1A
6	**C**	F	25.1	1.2	17.7***	CMT****
8	**D**	M	21.2	1.2	15.0*	CMT 1A
9	**E**	M	32.8	1.3	18.5	CMT 1A
9	**F**	F	30.9	1.3	17.2	CMT****
9	**G**	M	27.7	1.4	14.8*	CMT****
10	**H**	F	51.0	1.4	24.9***	CMT****
10	**I**	F	28.0	1.4	14.0*	CMT****
10	**J**	F	48.0	1.5	21.3**	CMT****
10	**K**	F	68.0	1.5	28.7***	CMT****
10	**L**	M	32.5	1.4	15.6*	CMT****
11	**M**	F	53.0	1.7	19.5	CMT****
11	**N**	M	30.1	1.3	17.5	CMT****
12	**O**	M	64.0	1.7	21.4**	CMT****
12	**P**	M	50.3	1.6	20.9	CMT****
13	**Q**	M	37.3	1.4	19.0	CMT****
14	**R**	F	46.4	1.6	19.1	CMT****
16	**S**	M	68.7	1.8	20.5	CMT****

* BMI - underweight; ** BMI - overweight; *** BMI - obesity; **** CMT subtype unspecified

The lower limb muscle strength, passive ROM, standing long jump test, and PBS scores obtained are shown in Table 2.

**Table 2:** Lower limb muscle strength, goniometry, long jump, and Pediatric Balance Scale (PBS) scores.

Participant	Age(years)	Muscle Strength (Kgf)												Goniometry (degrees)						Long Jump(cm)	PBS
		IR	IL	ER	EL	FPR	FPL	DFR	DFL	EKR	EKL	EHR	EHL	PFR	PFL	DR	DL	PAR	PAL		
A	6	6	5	7	6	17	20	6	5	12	8	13	13	50	42	10	12	190	145	38	55
B	6	4	4	7	6	14	14	7	6	9	10	13	10	65	60	20	20	145	155	60	54
C	6	2	3	2	2	6	8	0	0	14	10	10	12	45	45	10	10	150	140	49	51
D	8	4	6	5	4	18	20	8	5	12	10	14	11	40	45	22	20	140	150	102	55
E	9	6	7	6	7	9	10	9	7	16	17	13	11	50	40	10	0	154	150	115	55
F	9	7	3	4	5	7	10	2	1	17	18	19	17	50	50	10	10	150	150	99	54
G	9	4	5	7	5	21	18	11	9	14	14	16	17	50	45	20	20	155	140	113	56
H	10	8	9	7	10	18	18	7	6	10	10	9	11	32	36	8	10	134	132	59	56
I	10	11	9	7	8	13	15	10	8	24	22	14	13	35	40	22	22	130	140	18	53
J	10	12	12	13	11	19	22	12	12	20	20	16	18	34	34	20	18	145	140	94	56
K	10	4	5	2	2	12	14	0	0	18	19	16	15	50	52	-10	0	140	130	62	51
L	10	6	4	8	10	9	15	6	5	9	9	17	14	40	35	10	17	130	130	63	53
M	11	9	9	10	12	11	9	10	10	20	23	20	24	42	52	0	0	138	138	94	56
N	11	7	5	5	5	15	15	6	4	18	16	13	13	50	40	5	10	140	145	108	56
O	12	7	8	6	6	13	16	7	7	18	17	11	11	36	30	0	0	136	140	83	56
P	12	8	6	7	7	20	20	13	11	15	16	11	11	40	50	20	10	120	120	107	56
Q	13	5	4	5	6	18	10	3	2	11	10	10	10	40	50	10	10	150	136	60	56
R	14	9	12	3	5	24	20	2	2	25	19	14	12	50	40	5	10	128	142	88	55
S	16	12	12	8	9	22	22	26	20	29	28	29	29	50	50	15	12	155	150	180	56

IR= Inverter Right Foot, IL= Inverter Left Foot, ER= Evertor Right Foot, EL= Evertor Left Foot; FPR= Plantar flexor Right; FPE= Plantar flexor Left, DFR= Dorsiflexor Right; DFL= Dorsiflexor Left; EKR= Extensor Knee Right; EKL= Extensor Knee Left; EHR= Extensor Hip Right; EHL= Extensor Hip Left; PFR= Plantar Flexion Right; PFL= Plantar Flexion Left, DR= Dorsiflexion Right, DL= Dorsiflexion Left, PAR= Popliteal Angle Right, PAL= Popliteal Angle Left; 1^st^= First Trial; 2^nd^= Second Trial, 3^rd^= Third Trial; PBS= Pediatric Balance Scale

The isometric muscle strength values were not proportional to the age of the participants. The dorsiflexor, invertor, and evertor muscle groups presented the lowest isometric muscle strength values, with the dorsiflexion strength being zero in participants C and K.

For balance, which was determined using the PBS, high scores were observed for the participants with CMT (scores ranged between 51 and 56), indicating a good overall performance. However, considering the PBS items separately, the most challenging tasks were identified as follows: standing with eyes closed, standing with one foot in front, standing on one foot, retrieving an object from the ground, and reaching forward.

The ROM data indicated that bilateral ankle joint mobility was preserved except in three cases in which there was limitation (participants H, N, and R), with dorsiflexion being less than 10 degrees, and in three cases with lack of mobility (participants K, M, and O), with dorsiflexion being equal to or less than zero. The bilateral popliteal angle was preserved in most participants (except for values lower than 140°) (Table 2).

For the standing long jump test, there was no increase in performance with age, and the values from 7 (A, H, I, K, L, O, Q) of the 19 participants were lower than the values described as normative^16^ (Table 2).

### Correlations between PBS and lower limb muscle strength

The results of the Spearman test indicated a strong positive correlation between balance and the strength of the following muscle groups: right plantar flexors (r=0.61; p=0.01), right dorsiflexors (r=0.59; p=0.01), and left dorsiflexors (r=0.59; p=0.01) and a moderate correlation between balance and the strength of the following muscle groups: right invertors (r=0.44; p=0.06), left invertors (r=0.41; p=0.08), and right evertors (r=0.44; p=0.06) - Table 3.

**Table 3:** Spearman coefficient of correlation (rho) and p value for muscle strength in the lower limbs and the Pediatric Balance Scale (PBS).

Muscle Groups	Spearman correlation coefficient with balance(rho)	P value
Right Foot Invertor	0.44	0.06
Left Foot Invertor	0.41	0.08
Right Foot Evertor	0.44	0.06
Left Foot Evertor	0.38	0.10
Right Plantar Flexor	0.61	0.01
Left Plantar Flexor	0.38	0.11
Right Dorsiflexor	0.59	0.01
Left Dorsiflexor	0.59	0.01
Right Knee Extensor	0.15	0.54
Left Knee Extensor	0.20	0.41
Right Hip Extensor	–0.07	0.77
Left Hip Extensor	0.04	0.88

## Correlations between the standing long jump test and passive ROM of the lower limbs

The values obtained in the correlation of the standing long jump test with the ROM of the lower limbs showed a weak positive correlation between the ROM of right plantar flexion (r=0.20; p=0.41), left plantar flexion (r=0.12; p=0.61), and left popliteal angle (r=0.25; p=0.31). There was a weak negative correlation for left dorsiflexion (r=-0.15; p=0.54), and no correlation was found for right dorsiflexion (r=0.09; p=0.69) or right popliteal angle (r=0.00; p=1.00), as shown in Table 4. Therefore, the data indicated no correlation between ankle and knee joint ROM with muscle strength as demonstrated in the standing long jump test.

**Table 4:** Pearson coefficient of correlation ® for passive range of motion of the lower limbs and the long jump test.

Measured range of motion of lower limbs	Pearson correlation coefficient (r) with the long jump test	P value
Right Plantar Flexion	0.20	0.41
Left Plantar Flexion	0.12	0.61
Right Dorsiflexion	0.09	0.69
Left Dorsiflexion	–0.15	0.54
Right Popliteal Angle	0.00	1.00
Left Popliteal Angle	0.25	0.31

## Discussion

This study demonstrated that participants with CMT presented weakness in the following muscle groups: foot evertors, invertors, dorsiflexors and plantar flexors. With the exception of dorsiflexion, the ROMs were preserved. Overall, balance was preserved; however, there was a deficit in specific items of the PBS. The standing long jump test indicated that muscle strength was preserved in the majority of the participants, with certain exceptions.

Although, by definition, the sensorimotor impairment in CMT disease is symmetrical, variations in muscle strength, flexibility, and motor coordination have been observed. Thus, some correlations were found only for the strength and ROM of either the right or left side. These correlations suggest that the preserved strength of dorsiflexors and plantar flexors positively influenced performance in tasks that required balance. The ROMs obtained did not seem to have affected the muscle strength.

### Muscle strength and balance

Balance is an essential factor in the coordination of motor responses, movements, and postural adjustments. For balance to be effective, several factors, such as the vestibular system, proprioceptive information, visual perception, muscle strength, and joint flexibility need to operate efficiently and harmoniously in the body^17^. The muscles surrounding the ankle are essential to maintaining balance because they provide proprioceptive information and correct small postural oscillations, in addition to correcting possible destabilization through muscular torque, thereby regulating the center of gravity and keeping the center of mass located between the feet^18^. Typically, the natural history of various subtypes of CMT involves, among other manifestations, the progressive reduction of distal muscle strength, which can impair the maintenance of the center of mass over the base of support, both dynamically and statically^2^.

The ankle strategy is the most frequently used strategy to maintain balance, and it requires the preservation of the plantar flexor, dorsiflexor, evertor, and invertor muscle strength^19^. This strategy is more effective when perturbations to balance are slow and small and the supporting surface is firm (i.e., during static balance)^19^. The ankle dorsiflexion produced during the ankle strategy is crucial to maintaining balance after a destabilization because when the forefoot is lifted, a counter-movement force is created, which helps to re-balance the body^20^. Thus, the reduction in dorsiflexor muscle strength observed in the participants may explain the deficit found in the maintenance of static balance.

In the study, the participants presented data consistent with the data reported in the literature^2^^,^ 3 ^,^ 5, such as reduced muscle strength, especially in the evertor and dorsiflexor muscles and shortening of the plantar flexor muscles. A study conducted by Nyström et al.^21^ established reference values for lower limb isometric muscle strength according to the age and body weight of healthy participants. Thus, the data obtained in the current study were compared with the reference values obtained by Nyström et al.^21^ using the weight and height of the participants because the reference values by age could lead to misinterpretation. It was found that most participants with CMT presented with isometric muscular strength compatible with their body weight and height. Exceptions were found for the dorsiflexor muscles of participants C, E, and N. Normative data for comparison were not found for the foot invertor and evertor muscles nor for the plantar flexor muscles. However, it is worth noting that in 9 of the 19 participants, the muscle strength of invertors and evertors was lower than 5 KgF, which suggests a strength deficit in these muscle groups.

For the participants in this study, who presented with reduced distal muscle strength, the tasks involving static balance were more affected than the tasks involving dynamic balance because static postures required greater ROM and higher torque of the ankle musculature^22^.

The balance deficits found in the participants of this study were not disabling, considering that the PBS score was close to the maximum (between 51 and 56). Because several factors positively or negatively affect balance^17^, it is possible that mechanisms compensating for the distal muscle strength deficits were used (e.g., use of the hip strategy and upper limb assistance). Furthermore, proprioception and stabilization mechanisms, such as muscle stiffness, are key factors in the establishment of balance^23^. Anticipatory control is another factor that could have been triggered by the patients to obtain static and dynamic balance control^22^^,^ 23.

The positive correlation observed between the isometric muscle strength of the dorsiflexors, plantar flexors, evertors, and invertors with balance suggests that the maintenance of muscle strength of these muscle groups may positively affect balance. Ribeiro et al.^24^ associated ankle muscle strength with balance in the elderly and, similar to Sundermier et al.^25^, who evaluated children, corroborated the current study, concluding that plantar flexor and dorsiflexor strength was positively associated with balance.

### ROM and standing long jump

The ROM available for a joint can also be defined as flexibility, which is an important element of physical fitness^26^. Flexibility can be achieved by active muscle contraction, referred to as dynamic flexibility, or by passive motion caused by a force external to the joint. Gender, anthropometric measurements, body composition, genetic, and pathological characteristics, in addition to the growth and development processes^26^, all influence flexibility. The participants with CMT in this study presented with a joint ROM with relative flexibility and a preserved motion arc, which established a weak correlation with performance in the standing long jump test.

The standing long jump test results of the participants were compared to the normative data described by Condon and Cremin^16^, who studied this variable in 534 children aged 4 to 15 years. The age-matched comparison with participants of the current study demonstrated that 7 (A, H, I, K, L, O, Q) of the 19 participants presented lower values than were described as normative.

During the performance of the standing long jump test, the additional impulse imparted to the jump by the swinging of the arms might have increase the distance jumped and the takeoff speed^27^. In this study, all participants were instructed to perform the test movement using the technique of propelling themselves with the arms. Ashbya and Heegaard^27^ indicated that the arm swing enhanced the force-producing ability of the lower extremity extensor muscles, slowing the contraction speed at key moments in the jump. To maintain balance throughout the jump, measures such as anticipatory control or even the employment of counterproductive mechanisms that reduce the jumping distance with free arm motion may have been adopted^27^. Considering that children with CMT are aware of their balance deficits, it is possible that they adopted anticipatory control measures using the free arms. Thus, the restricted use of the arms by certain participants may explain in part the lower jumping performance of participants A, H, I, K, L, O and Q, which was significantly lower than the overall mean of the jumps considered.

The standing long jump test, while considered a motor task or skill, is a complex motor pattern that requires the coordinated performance of all body segments, where the impulse and the landing must be performed with both feet. The horizontal jump measures explosive force, is strongly correlated with isokinetic measures of lower limb strength, and is indicated as a good predictor of performance in the standing long jump^10^.

The lack of correlation or even the weak correlation found between ROM and the standing long jump test may be attributed to the fact that most participants in this study had relatively preserved ROM in distal muscles. A group of affected participants without preserved ROM should be evaluated to assess the influence of passive ROM on the standing long jump, which was a limitation of the present study.

The sample size, heterogeneity of the CMT subtypes, different levels of motor maturation, and different anthropometric characteristics are common limitations in studies of this nature. Based on anthropometric data, the participants were classified in all categories of BMI, and 21% of them were obese, which may have influenced the results. BMI does not seem to negatively affect flexibility, in contrast to propulsion tests^28^. Obese individuals suffer disadvantages in more challenging balance activities, such as standing on one foot^29^. For muscle strength, a recent review^30^ indicated that although obese individuals presented with higher absolute values compared to their normal-weight peers, obesity had no impact on the intrinsic properties of the muscle to generate force. Thus, the interference of BMI on the data obtained in the present study was considered minimal.

The results of this study may assist the physical therapist in making decisions during clinical practice because they suggest that preserved dorsiflexor and plantar flexor muscle strength is associated with better static and dynamic balance performance. Similarly, the maintenance and/or gain of joint mobility, especially of dorsiflexion through stretching, may promote good functional performance and muscle strength as demonstrated in the standing long jump test. Thus, in the treatment of children and adolescents with CMT disease, the maintenance and/or gain of strength and flexibility of the dorsiflexor and plantar flexor muscles should be prioritized.

## Conclusion

The maintenance of distal muscle strength in children with CMT contributes to their performance of balance tasks. The losses found in passive ROM of the lower limbs seem not to have been sufficient to affect muscle strength in the horizontal long jump.
